# Commentary on Getty *et al*.: Complementarity of harm reduction and contingency management

**DOI:** 10.1111/add.70316

**Published:** 2026-01-12

**Authors:** John M. Roll

**Affiliations:** ^1^ Community and Behavioral Health Washington State University Spokane WA USA

**Keywords:** cocaine, contingency management, harm reduction, methamphetamine treatment, patient care, service provision

*Both harm reduction and contingency management approaches have the potential to improve patient lives. Supporting both, depending on patient attributes and desires, is an ethical approach to helping those in treatment achieve their recovery goals*.

It is a pleasure to add a brief commentary to the article by Getty and colleagues [1]. One area of concern noted by the authors is the belief held by some that contingency management (CM) and harm reduction approaches are incompatible. It is this perception to which I will devote this commentary.

Harm reduction is a powerful and effective therapeutic approach/philosophy that seeks to minimize substance‐related harm while enhancing quality of life [2]. Harm reduction embraces a patient‐driven approach to substance use management. This is not unique to substance use disorder as it has been argued that optimal, patient‐driven health should be a therapeutic goal for other health conditions as well [3]. CM for substance use disorder treatment is an efficacious behavioral approach that uses reinforcement to encourage behavior change [4]. CM allows patients and providers to celebrate therapeutic success, and this may foster an enhanced therapeutic alliance relative to treatment approaches that focus on the negative aspects of relapse. Patients and their loved ones should generally be given as much agency as possible in selecting the path that best suits their current recovery goals and environments. It would be appropriate for a well‐funded healthcare system to offer multiple options, including both harm reduction and CM approaches.

A central tenet of concern related to CM by some in the harm reduction community is that CM programs focused on reducing substance use employ negative urine tests as the primary measure. The belief that this focus on abstinence as a goal could undermine other therapeutic behaviors in which the patient might engage is a valid concern, but one, we believe, can be satisfactorily addressed through clinical flexibility and appreciation of the patient's unique attributes.

CM is not simply an abstinence‐oriented intervention that patients choose to participate in. CM is an approach that can be applied to most observable behavior to alter the frequency of that behavior. This can include everything from supporting a child in doing their homework, to encouraging recycling, to treating methamphetamine use disorder. Although it is true that when we discuss CM in a substance use treatment context, it almost exclusively refers to an abstinence‐based approach to treating psychostimulant use disorder, that does not need to be the sole domain of the CM interventions.

Relevant examples include not only CM for drug abstinence [5], but reductions in drug use [6], attendance [7], job seeking [8], improvements in general health behavior [9] and medication compliance [10]. There is no a priori reason why CM cannot be applied to behaviors identified via patient engagement in a harm reduction program. For example, suppose a patient and their support team want to engage in safer substance use practices. In that case, there is no reason why that effort could not be supported by a CM intervention that reinforces behaviors such as reducing the amount and frequency of use, use of sterile supplies and the avoidance of using drugs alone.

Patient choice is central to harm reduction philosophy and success. This implies that recovery goals can change over time, and as they do, therapeutic approaches should also be flexible [11]. For example, a person in a harm reduction program who started out focusing on safer drug use may decide at some point that they wish to pursue reductions or cessation of use. If that happens, an ethical choice is to offer them the best evidence‐based approach to achieving that goal, and for psychostimulants that is CM, which is focused on non‐use [5]. The opposite may also be true for those who engage in a CM program without submitting any stimulant negative urine tests. That is, they should be able to take part in a harm reduction approach as an ethical alternative.

As researchers and clinicians, we cannot avoid bringing our biases to our work. However, as our field evolves, I believe it is important to recognize that each patient brings unique attributes to a potential therapeutic interaction. Those attributes should be heavily weighted in helping them select the best therapeutic alternative at any given time. Perhaps there should be no hierarchy of evidence‐based treatments. Instead, a decision about the type and nature of an evidence‐based approach should be based on a shared decision‐making process involving those seeking treatment, their loved ones and providers. If we consider a patient's circumstances at a given time and acknowledge that circumstances can change, there will not always be one correct approach. By using the diversity of treatment options available, we maximize the likelihood of saving a patient's life.

## AUTHOR CONTRIBUTIONS

**John M. Roll:** Conceptualization (equal); methodology (equal); resources (equal).

## DECLARATION OF INTERESTS

None.

## Data Availability

NA.

## References

[1] Getty C‐A , McQuarrie T , Brobbin E . Contingency management interventions for substance use and addictive behaviours: Review of the UK evidence base. Addiction. 2026;121(4):729–746. 10.1111/add.70240 41242967 PMC12980297

[2] Collins SE , Clifasefi SL . Harm reduction treatment for substance use. In: Advances in Psychotherapy‐Evidence Based Practice 49; 2023.

[3] Roll JM . Focus on optimal health, not ideal health. Patient Experience Journal. 2021; 8(3):22–23. 10.35680/2372-0247.1639

[4] Rash CJ , McDonell M , Erath T , parent S , Wattenberg S . A call to action: Evidence‐based contingency management. Am J Psychiatry. 2025 182(7): 599–602. 10.1176/appi.ajp.20240261 40134267

[5] Prendergast M , Podus D , Finney J , Greenwell L , Roll J . Contingency management for treatment of substance use disorders: a meta‐analysis. Addiction. 2006;101(11):1546–1560. 10.1111/j.1360-0443.2006.01581.x 17034434

[6] Preston KL , Umbricht A , Wong CJ , Epstein DH . Shaping cocaine abstinence by successive approximation. J Consult Clin Psychol. 2001 Aug;69(4):643–654. 10.1037/0022-006X.69.4.643 11550730

[7] Pfund RA , Ginley MK , Rash CJ , Zajac K . Contingency management for treatment attendance: a meta‐analysis. J Subst Abuse Treat. 2022 Feb;133:108556. 10.1016/j.jsat.2021.108556 34210566 PMC8702584

[8] Wong CJ , Dillon EM , Sylvest CE , Silverman K . Contingency management of reliable attendance of chronically unemployed substance abusers in a therapeutic workplace. Exp Clin Psychopharmacol. 2004 Feb;12(1):39–46. 10.1037/1064-1297.12.1.39 14769098

[9] Erath TG , DiGennaro Reed FD . Technology‐based contingency management for walking to prevent prolonged periods of workday sitting. J Appl Behav Anal. 2022;55(3):746–762. 10.1002/jaba.917 35388466

[10] DeFulio A , Silverman K . The use of incentives to reinforce medication adherence. Prev Med. 2012;55(Supplement, 1):S86–S94. 22580095 10.1016/j.ypmed.2012.04.017 PMC3424340

[11] Khazanov K , McKay JR , Rawson R . Should contingency management protocols and dissemination practices be modified to accommodate rising stimulant use and harm reduction frameworks? Addiction. 2024;119(9):1505–1514. 10.1111/add.16497 38627885

